# Discovery of Strong 3-Nitro-2-Phenyl-*2H*-Chromene Analogues as Antitrypanosomal Agents and Inhibitors of *Trypanosoma cruzi* Glucokinase

**DOI:** 10.3390/ijms25084319

**Published:** 2024-04-13

**Authors:** Shane M. Carey, Destiny M. O’Neill, Garrett B. Conner, Julian Sherman, Ana Rodriguez, Edward L. D’Antonio

**Affiliations:** 1 Department of Natural Sciences, University of South Carolina Beaufort, 1 University Boulevard, Bluffton, SC 29909, USA; 2 Department of Microbiology, New York University School of Medicine, 430 East 29th Street, New York, NY 10016, USAana.rodriguezfernandez@nyulangone.org (A.R.)

**Keywords:** Chagas disease, drug discovery, glucokinase, glucose kinases, hexokinase, human African trypanosomiasis, inhibitors, neglected tropical diseases, 3-nitro-2-phenyl-*2H*-chromene analogues

## Abstract

Chagas disease is one of the world’s neglected tropical diseases, caused by the human pathogenic protozoan parasite *Trypanosoma cruzi*. There is currently a lack of effective and tolerable clinically available therapeutics to treat this life-threatening illness and the discovery of modern alternative options is an urgent matter. *T. cruzi* glucokinase (*Tc*GlcK) is a potential drug target because its product, d-glucose-6-phosphate, serves as a key metabolite in the pentose phosphate pathway, glycolysis, and gluconeogenesis. In 2019, we identified a novel cluster of *Tc*GlcK inhibitors that also exhibited anti-*T. cruzi* efficacy called the 3-nitro-2-phenyl-2*H*-chromene analogues. This was achieved by performing a target-based high-throughput screening (HTS) campaign of 13,040 compounds. The selection criteria were based on first determining which compounds strongly inhibited *Tc*GlcK in a primary screen, followed by establishing on-target confirmed hits from a confirmatory assay. Compounds that exhibited notable in vitro trypanocidal activity over the *T. cruzi* infective form (trypomastigotes and intracellular amastigotes) co-cultured in NIH-3T3 mammalian host cells, as well as having revealed low NIH-3T3 cytotoxicity, were further considered. Compounds *GLK2-003* and *GLK2-004* were determined to inhibit *Tc*GlcK quite well with IC_50_ values of 6.1 µM and 4.8 µM, respectively. Illuminated by these findings, we herein screened a small compound library consisting of thirteen commercially available 3-nitro-2-phenyl-2*H*-chromene analogues, two of which were *GLK2-003* and *GLK2-004* (compounds **1** and **9**, respectively). Twelve of these compounds had a one-point change from the chemical structure of *GLK2-003*. The analogues were run through a similar primary screening and confirmatory assay protocol to our previous HTS campaign. Subsequently, three in vitro biological assays were performed where compounds were screened against (a) *T. cruzi* (Tulahuen strain) infective form co-cultured within NIH-3T3 cells, (b) *T. brucei brucei* (427 strain) bloodstream form, and (c) NIH-3T3 host cells alone. We report on the *Tc*GlcK inhibitor constant determinations, mode of enzyme inhibition, in vitro antitrypanosomal IC_50_ determinations, and an assessment of structure–activity relationships. Our results reveal that the 3-nitro-2-phenyl-*2H*-chromene scaffold holds promise and can be further optimized for both Chagas disease and human African trypanosomiasis early-stage drug discovery research.

## 1. Introduction

Chagas disease (or American trypanosomiasis) is a neglected tropical disease caused by the pathogenic protozoan parasite *Trypanosoma cruzi,* which is frequently found in two hosts: humans and various vector-borne triatomine insects. Transmission principally occurs when a *T. cruzi*-infected triatomine insect, such as *Triatoma sanguisuga* or *Triatoma gerstaeckeri*, among other similar insects, bites a human host [1,2]. During this process, the insect feces are incidentally mixed into the bite wound and *T. cruzi* parasites pass into the host’s bloodstream [2,3]. The disease was originally discovered in 1909 by the Brazilian physician Carlos Chagas [4] and it is estimated to affect 6–7 million people annually [3]. The regions of the world with the most reported cases are the neglected rural tropics of Latin America. The severity and outcomes of the infection may vary due to factors such as the age of the individual (at the time of infection), the site of the infection, as well as the strain of *T. cruzi*. Chagas disease is also presents in the forms of acute and chronic stages of infection. During the acute stage, which lasts about two months, parasites are found circulating within the bloodstream of the affected individual [2]. This initial stage may be asymptomatic or carry mild symptoms, such as a fever or swelling at the site of inoculation [3]. The chronic stage exhibits mostly asymptomatic behavior, and because of this, most affected individuals are unaware of their infection. However, 20–30% of those in the chronic stage may experience severe and life-threatening medical conditions, including one or more of the following: arrhythmic heartbeat abnormalities or dilation of the esophagus, colon, brain, and/or heart [3,4,5,6].

Only two drugs, benznidazole and nifurtimox, are currently approved for the treatment of Chagas disease [3,7,8,9]. These therapeutics are effective in treating the infections; however, they lead to adverse reactions and poor patient compliance [10,11]. Both therapeutic drugs are highly effective if they are given at the onset of the acute stage, but they may not be effective during the chronic stage [12,13]. Efficacy of the treatments decreases when a person has been infected for a longer duration of time; the risk of adverse effects also increases, with up to 40% of treated adults and the elderly experiencing these harmful effects [3]. The side effects of these therapeutics include anorexia, nausea, peripheral neuropathy, insomnia, and many others [8,9,14]. Benznidazole and nifurtimox each have a surplus of drawbacks and there have not been any successful alternatives to these therapeutics since their inception over 55 years ago [11]. Accordingly, the early-stage therapeutic drug discovery research focused on this neglected tropical disease is a high priority.

**Figure 1:**
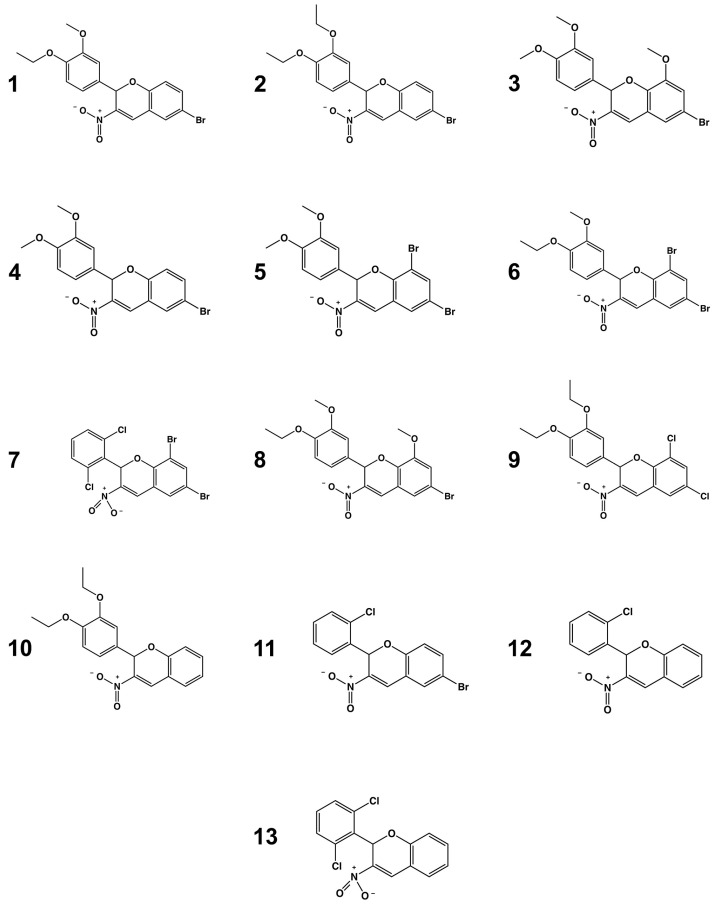
Library of 3-nitro-2-phenyl-*2H*-chromene analogues (**1**–**13**). For systematic names and SMILES codes of the compounds, please refer to Tables S1 and S2 in the Supplementary Information section, respectively.

*T. cruzi* hexokinase (*Tc*HxK) and glucokinase (*Tc*GlcK) are ATP-dependent glycolytic enzymes involved in energy metabolism of the parasite [15,16]. Their key role is in the production of d-glucose-6-phosphate (G6P), which is a metabolite positioned between glycolysis, gluconeogenesis, and the pentose phosphate pathway (PPP) [7,17,18]. Two life stages are commonly observed during human infection: the trypomastigote and the intracellular amastigote [5]. Moreover, critically important to the survival of these life stages is the requirement that these pathways remain operational [18,19,20]. The PPP produces NADPH that has a role in maintaining antioxidant levels within the *T. cruzi* cell; it is also responsible for the production of ribose-5-phosphate that allows for the de novo synthesis of purines and pyrimidines, as precursors for nucleotides/nucleic acids [21,22]. Glycolysis has also been investigated for its importance in *T. cruzi* [19,23,24]. As a consequence, these metabolic pathways have been targeted for the early-stage therapeutic drug discovery for Chagas disease. The concept of limiting the flux of G6P by inhibiting either *Tc*HxK and/or *Tc*GlcK to cause *T. cruzi* cell death appears to be a good tactic and it is one on which our laboratory has focused for the past decade. *Tc*GlcK has been recognized as a potential drug target based on various studies involving small-molecule inhibitors and natural products [17,25,26,27]. We recently discovered that the 3-nitro-2-phenyl-*2H*-chromene compound class, via a high-throughput screening (HTS) campaign of 13,040 compounds, includes two antichagasic lead compounds [17]. These compounds were designated as *GLK2-003* and *GLK2-004*, but in this work they were named as compounds **1** and **9**, respectively (Figure 1). The acceptance criteria for hits and leads in *T. cruzi* drug discovery pipelines have previously been discussed [28,29]. Moreover, this finding has increased our interest in exploring the inhibitory activity of commercially available 3-nitro-2-phenyl-*2H*-chromene compounds against *Tc*GlcK and *T. cruzi* parasites co-cultured in mammalian cells. With the highly plausible postulation about the mechanism of simultaneous inhibition of *Tc*GlcK and *Tc*HxK by the 3-nitro-2-phenyl-*2H*-chromene compound class [17,30], we were eager to learn of any observable trends in this regard. We were also interested in learning if these compounds would impart a growth inhibitory effect on *T. brucei*, the etiological agent of human African trypanosomiasis [31].

The work herein focused on performing biochemical and biological evaluations on thirteen 3-nitro-2-phenyl-*2H*-chromene analogues (Figure 1). This library of compounds was screened against *Tc*GlcK, measuring inhibitor constant (K_i_) values to assess enzyme-inhibition potency. We conducted in vitro phenotypic screens of the compounds against the trypanosomatid parasites *T. cruzi* and *T. brucei*. A cytotoxicity assessment was also performed on the NIH-3T3 fibroblasts. A structure–activity relationship (SAR) analysis was devised to help understand the molecular determinants pertaining to the 3-nitro-2-phenyl-*2H*-chromene scaffold that gave rise to the improved/worsened *Tc*GlcK enzyme inhibition and parasite growth inhibition.

## 2. Results and Discussion

### 2.1. TcGlcK Primary Screen, Confirmatory Assay, and T. cruzi Biological Assay

**Figure 2:**
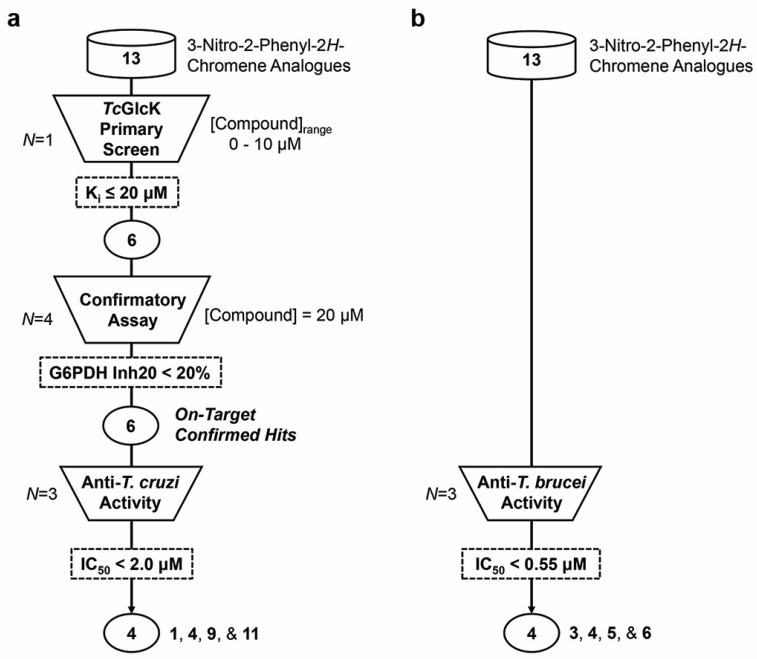
Flowchart diagrams with assay checkpoints (trapezoids) and compound filters (dashed rectangles) to narrow down top-performing compounds from a 13-compound library of 3-nitro-2-phenyl-*2H*-chromene analogues (cylinders). Compounds were screened against (**a**) *Tc*GlcK/*T. cruzi* parasites and (**b**) *T. brucei* parasites. *N* indicates the number of times an assay was performed; *N* = 1 for the *Tc*GlcK primary screen, *N* = 4 for the confirmatory assay, and *N* = 3 for the anti-*T. cruzi* and anti-*T. brucei* activity assays.

Enzyme inhibition assays were performed in the primary screen for compounds **1**–**13** versus *Tc*GlcK from which K_i_ values were determined (*N* = 1) for each compound using Dixon plots (Table 1). Compounds that resulted in a K_i_ > 20 µM were excluded from further analysis. Compounds **1**, **4**, **6**, **9**, **11**, and **12** continued through the pipeline, as shown in Figure 2a; the next filter, the confirmatory assay, involved a similar process. The purpose of the confirmatory assay was to test if *Lm*G6PDH (the enzyme-coupled reaction) was being inhibited by the compounds by more than 20%, and if so, they were removed from consideration. The main difference was that *Tc*GlcK, d-glucose, and ATP were removed from the assay and the starting substrates were G6P and NADP^+^. From quadruplicate independent measurements (*N* = 4), we did not observe any significant *Lm*G6PDH inhibitory effects, and thus, compounds **1**, **4**, **6**, **9**, **11**, and **12** continued onto the *T. cruzi* biological assay and were defined as on-target confirmed hits. For completeness, compounds **1**–**13** were all tested in vitro against the *T. cruzi* infective form (e.g., trypomastigotes and intracellular amastigotes) co-cultured in mammalian NIH-3T3 fibroblasts, in triplicate (*N* = 3) (Table 1). We observed low micromolar or better efficacy against *T. cruzi* for most compounds. Compounds that revealed an IC_50_ ≥ 2.0 μM were filtered out of the pipeline; in this case, compounds **6** and **12** were rejected. The leading 3-nitro-2-phenyl-2*H*-chromene analogues were **1**, **4**, **9**, and **11**. These compounds had similar or better antichagasic potency (see Table 1) compared to the growth inhibition effect observed in *T. cruzi* exposed to benznidazole (control). Benznidazole was observed to have an IC_50_ of 1.12 ± 0.095 μM in our previous work [27].

**Figure 3:**
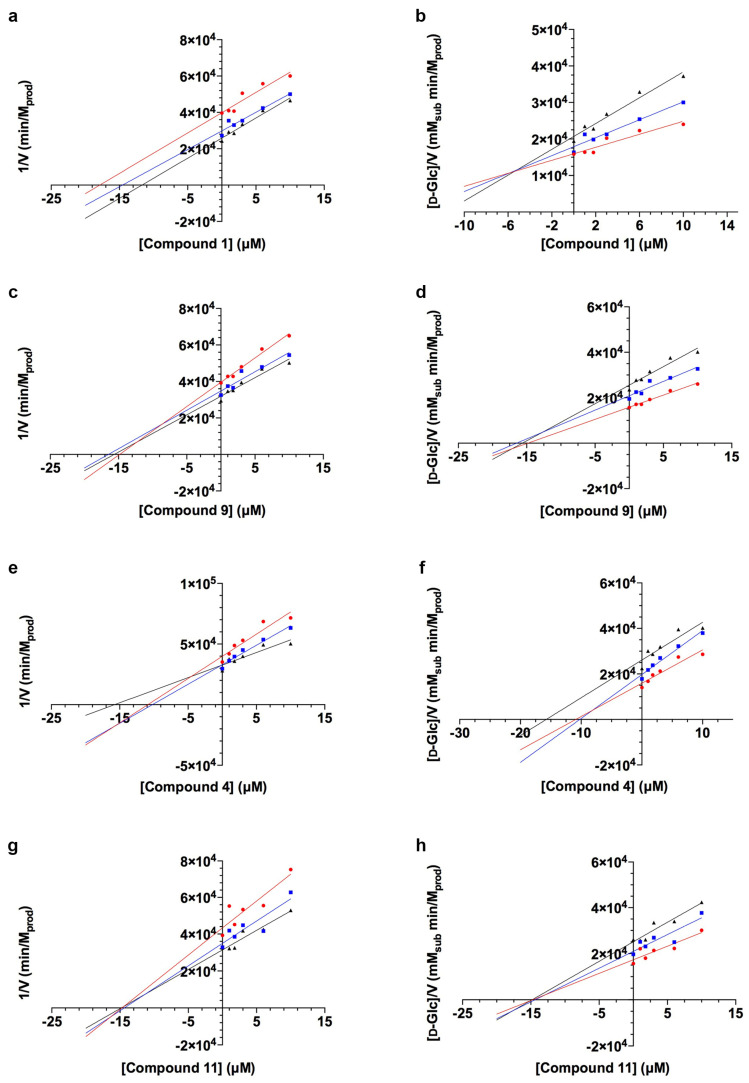
Enzyme-inhibitor kinetics for *Tc*GlcK and compounds **1** (*GLK2-003*), **9** (*GLK2-004*), **4**, and **11** in order to determine K_i_ values. Panels (**a**,**c**,**e**,**g**) represent Dixon plots of 1/V as a function of inhibitor concentration. Values calculated from the extrapolation of the intersection of the three lines with the *X*-axis were as follows: K_i_ = 6.2 ± 1.2 μM for compound **1**, K_i_ = 12.3 ± 5.5 μM for compound **9**, K_i_ = 5.5 ± 1.8 μM for compound **4**, and K_i_ = 20.0 ± 4.3 μM for compound **11**. Panels (**b**,**d**,**f**,**h**) represent a second set of Dixon plots characterized as [d-Glc]/V as a function of inhibitor concentration, which were used to determine the mode of inhibition. The modes of inhibition were determined as uncompetitive inhibition for compound **1**, mixed-mode inhibition for compounds **9** and **4**, and noncompetitive inhibition for compound **11**. The inhibitor concentrations ranged from 0.0–10.0 μM at three different substrate concentrations, 0.4 mM d-Glc (red circles), 0.6 mM d-Glc (blue squares), and 0.8 mM d-Glc (black triangles). In all panels, one representative experiment is displayed but three independent experiments were performed. Dixon plot graphs corresponding to an individual compound are from the same experiment.

A thorough determination of *Tc*GlcK K_i_ values was performed for these particular compounds, in which two more independent enzyme inhibition experiments were conducted to have a total of three replicates. The *Tc*GlcK enzyme–inhibitor K_i_ values of these lead compounds are shown in Table 1 and their corresponding Dixon plots are displayed in Figure 3. Relative standard deviations observed for the K_i_ values were slightly higher than desired for each of the four compounds (**1**, **4**, **9**, and **11**), which ranged from 19–45%; we suspect it arose due to random error in the assay with many components being added per reaction. The mode of inhibition was determined through interpretation of these plots using the Cornish–Bowden method [32]. Briefly, uncompetitive inhibition was observed for compound **1** (*GLK2-003*), mixed-mode inhibition was observed for compounds **4** and **9** (*GLK2-004*), and noncompetitive inhibition was observed for compound **11**.

**Figure 4:**
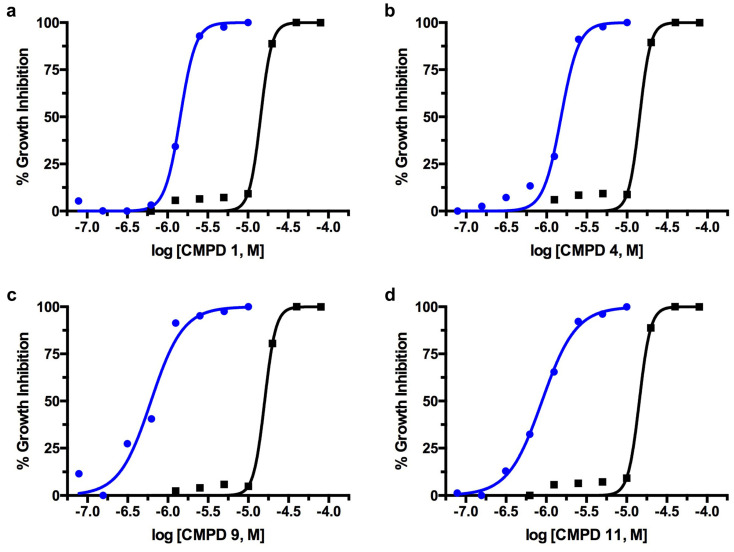
The in vitro dose-response curves of compound activity on *T. cruzi* (Tulahuen strain) infective form co-cultured in NIH-3T3 fibroblasts (blue) in comparison to NIH-3T3 fibroblast cytotoxicity (black) for compounds (**a**) **1**, (**b**) **4**, (**c**) **9**, and (**d**) **11**.

The in vitro dose-response IC_50_ plots of compound activity against *T. cruzi* are presented in Figure 4 for compounds **1**, **4**, **9**, and **11**. All of the investigational compounds exhibited higher potency against *T. cruzi* in culture compared to enzymatic assays against *Tc*GlcK. In particular, the ratio of *Tc*GlcK K_i_/*T. cruzi* IC_50_ was observed to be eight-fold (or more) in 9 out of the 13 compounds (please refer to Table 1). The apparent loss of correlation between phenotypic IC_50_ values and enzyme–inhibitor K_i_ values indicates an off-target effect. This observation is consistent with our previously proposed hypothesis that the 3-nitro-2-phenyl-*2H*-chromene class of compounds is likely to inhibit both *Tc*GlcK and *Tc*HxK simultaneously [17]. Thus, we suspect the off-target effect is due to *Tc*HxK. In our previous study on d-glucosamine–analogue inhibitors of *Tc*GlcK, we observed a similar trend and magnitude of the ratio of *Tc*GlcK K_i_/*T. cruzi* IC_50_. For example, carboxybenzyl glucosamine was the strongest competitive inhibitor of *Tc*GlcK in the study with a reported K_i_ of 0.71 μM, but it exhibited an in vitro percent-growth-inhibition IC_50_ of 48.73 μM in *T. cruzi* culture. This magnitude of a 68.6-fold differential led us to conclude a loss of correlation [27]. It is now even more apparent that there is a need to investigate *Tc*HxK experimentally to confirm whether it is indeed acting as the second target in *Tc*GlcK medicinal chemistry studies.

Regarding the 3-nitro-2-phenyl-*2H*-chromene class of compounds inhibiting the *T. cruzi* glucose kinases, Omolabi and colleagues reported on a computational investigation where they predicted the ligand-binding sites in *Tc*GlcK and *Tc*HxK [30]. For the *Tc*GlcK binding site, they showed that compounds **1** (*GLK2-003*) and **9** (*GLK2-004*) had an affinity to interact with residues P92 and T185, which were found near the d-glucose binding site, as visualized in the solved X-ray crystal structure of *Tc*GlcK first reported by Cordeiro and colleagues [33]. They also pointed out that in *Tc*HxK, residues of interest were P163 and T237, which were also found near the d-glucose binding site [30]. Compounds **1** and **9** would be considered as competitive inhibitors in this case; however, our study showed a different outcome. Compounds **1** and **9** exhibited uncompetitive and mixed-mode inhibition, respectively. We cannot explain the difference in the enzyme kinetics to the computational study at the present time, but we can suggest an alternative experimental method to study the 3-nitro-2-phenyl-*2H*-chromene binding site interaction of *Tc*GlcK with compounds **1** and/or **9** (and others of the class). It appears that the best method would be X-ray crystallography since there are many published structures available in the protein databank for *Tc*GlcK, including structures of *Tc*GlcK—inhibitor complexes [25,27,33]. Circling back to the key residues of inhibitor interactions proposed by Omolabi et al. (*vide supra*), we recently reported a structural superposition between the X-ray crystal structure of *Tc*GlcK (PDB entry 7S2H) and the AlphaFold computed structure model of *Tc*HxK (AlphaFold DB entry AF-Q8ST54-F1) [25]. This allowed for the development of a starting framework to identify the location of these residues; our laboratory is pursuing testing to determine whether they have a key role in the proposed inhibitor binding sites. Structural insight can also be gained by comparing solved hexokinase structures of other parasitic protozoa, such as *Plasmodium vivax* hexokinase (PDB entry 6VYG) [34] and *Plasmodium falciparum* hexokinase (PDB entry 7ZZI) [35].

### 2.2. T. brucei Biological Assay

**Figure 5:**
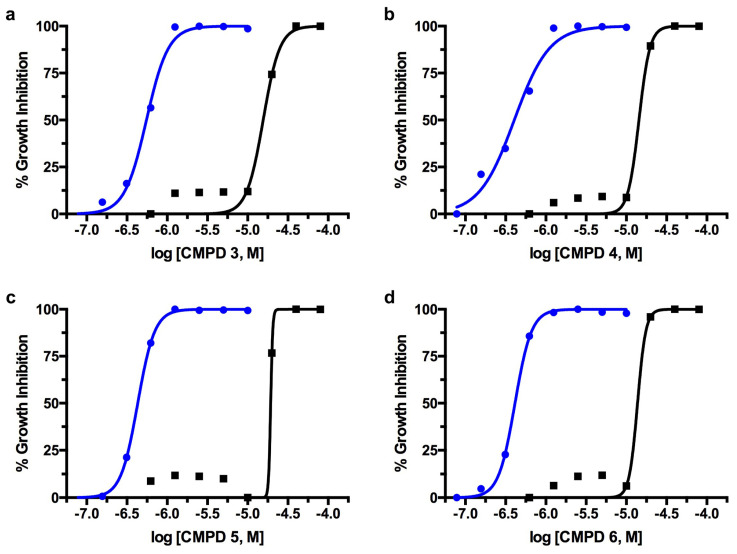
The in vitro dose-response curves of compound activity on *T. brucei brucei* (427 strain) bloodstream form (blue) in comparison to NIH-3T3 fibroblast cytotoxicity (black) for the high-performing compounds (**a**) **3**, (**b**) **4**, (**c**) **5**, and (**d**) **6**.

Compounds **1**–**13** were all tested against another trypanosomatid parasite, *T. brucei brucei* (427 strain) bloodstream form (Figure 2b and Table 1). With regards to anti-*T. brucei* activity, which was more efficacious, nine compounds (**1**, **3**, **4**, **5**, **6**, **7**, **8**, **9**, and **11**) exhibited nanomolar potency. All thirteen compounds exhibited significant growth inhibition of the *T. brucei* bloodstream form, with compounds **3**, **4**, **5**, and **6** being the most potent (Figure 5). Interestingly, three compounds were identified as effective dual inhibitors of *T. cruzi* and *T. brucei*, namely **5**, **7**, and **9**. Although, compounds **5** and **7** lacked the necessary strong binding affinity for *Tc*GlcK. Observing this enhanced growth inhibition against *T. brucei* is justified, since Chambers and colleagues determined that *T. brucei* hexokinase 1 is essential to the bloodstream form, as demonstrated by RNA interference [36]. Since there are two glucose kinases in *T. brucei*, *T. brucei* hexokinase 1 and *T. brucei* hexokinase 2, they are most likely being inhibited in the same manner as the glucose kinases in *T. cruzi*. Inhibitor design and development, which could rely on *Tc*GlcK as a surrogate model for the glucose kinases of *T. brucei*, seems to be a compelling strategy for early-stage therapeutic drug discovery and more so now that AlphaFold has produced a computed structural model for *T. brucei* hexokinase 1 (see AlphaFold DB entry AF-Q95PL2-F1 on the AlphaFold Protein Structure Database) [37,38].

### 2.3. Mammalian Cytotoxicity Assay

To assess the toxicity of the compounds involved in this study against mammalian NIH-3T3 fibroblasts, cell cultures were grown in the absence of parasites and were exposed to the 3-nitro-2-phenyl-*2H*-chromene analogues. At various concentrations, compounds **1**–**12** each produced a TC_50_ plot from independent experiments performed in triplicate (*N* = 3) (Table 1). We were unable to acquire a good quality S-shaped TC_50_ curve for compound **13** and its TC_50_ value was not determined. Compounds **1**–**10** and **12** exhibited TC_50_ values ranging from 9.45–26.1 μM; compound **11** resulted in having the most cytotoxic effect, with a TC_50_ value of 4.12 μM. A therapeutic window exists amongst the leading compounds identified against *T. cruzi* (Figure 4) and *T. brucei* (Figure 5) with respect to cytotoxicity activity against NIH-3T3 fibroblasts.

### 2.4. Structure–Activity Relationship Analysis

**Figure 6:**
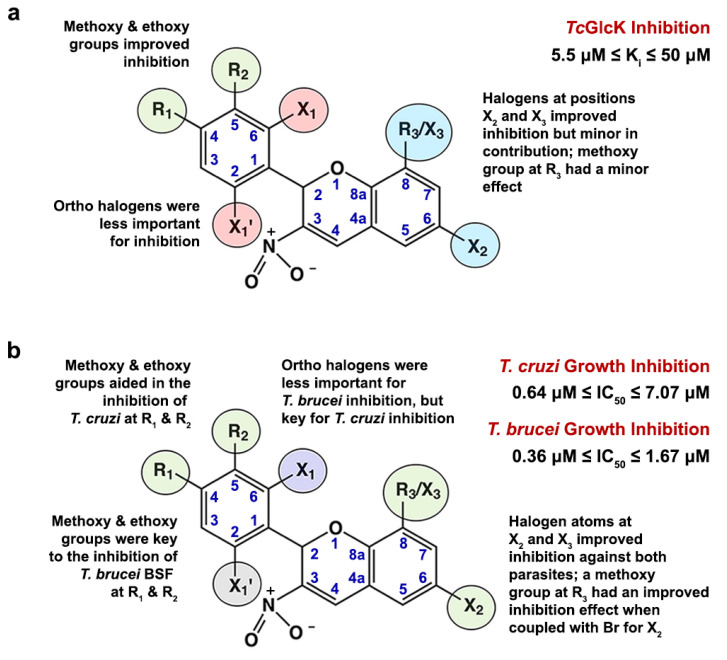
Structure–activity relationships observed from the 3-nitro-2-phenyl-*2H*-chromene scaffold representing the thirteen analogues. Substituent effects are highlighted for (**a**) *Tc*GlcK inhibition and (**b**) in vitro growth inhibition of trypanosomatid parasites *T. cruzi* and *T. brucei*. An atom numbering system is provided for the scaffold in blue.

An SAR analysis for the 3-nitro-2-phenyl-2*H*-chromene analogues observed in this study was performed using the software DataWarrior, version 5.5.0 [39]. The program was arranged to perform an R-group deconvolution in order to produce an SAR analysis tabular display. This was achieved by first loading a list of compounds **1**–**13** as SMILES codes (see Table S2 in the Supplementary Information section). Second, an automatic SAR analysis was implemented, selecting the scaffold type as the most central ring system. In addition to the chemical structures, input files also contained information pertaining to K_i_ values for *Tc*GlcK, IC_50_ values for *T. cruzi* and *T. brucei* parasites, and TC_50_ values for NIH-3T3 cells. In the case of *Tc*GlcK inhibition (Figure 6a), substituents R_1_ and R_2_ positioned on atoms C4 and C5 of the phenyl ring, respectively, revealed that ethoxy and/or methoxy groups were important for strengthened inhibition. This trend was noted in the stronger *Tc*GlcK inhibitors, compounds **1**, **4**, **6**, **9**, and **10**. Moreover, when a compound contained an ethoxy group for R_1_ and a methoxy group for R_2_ (concurrently), this arrangement was optimal for stronger *Tc*GlcK inhibition (e.g., compounds **1** and **6**). Halogen atoms at the ortho position(s) on the phenyl group bound to atoms C6 and C2 were designated as X_1_ and X_1_^′^, respectively. These substituents were considered less important for the inhibition of *Tc*GlcK, as demonstrated by compounds **7** and **13**. Substituents found on the *2H*-chromene ring system at carbon atoms C6 and C8 improved *Tc*GlcK inhibition when arranged as a pair of halogen atoms (e.g., a bromine atom for X_2_ and a bromine atom for X_3_), with compounds **6** and **9** as representative examples. However, positions X_2_ and X_3_/R_3_ are not major contributors to the inhibitory effect of *Tc*GlcK, since some of the weaker compounds, such as compounds **5** and **7**, also had this arrangement of halogen atoms. Compounds containing a methoxy group at position R_3_, such as compounds **3** and **8**, also contributed to a minor inhibitory effect on *Tc*GlcK. With a focus on compounds **1**, **4**, and **9**, which have methoxy and/or ethoxy substituents at R_1_ and R_2_ (see Figure 6a), it is unclear at the present time why the modes of inhibition are uncompetitive for compound **1** and mixed-mode for compounds **4** and **9**; we expected all three of these compounds to exhibit a similar style of inhibition. Finally, we observed noncompetitive inhibition by compound **11**, but the substituent pattern was not the same as compounds **1**, **4**, and **9**. It is crucial to solve the crystal structures of *Tc*GlcK—inhibitor complexes to identify the binding site(s) for these compounds and to understand the nature of their inhibition modes as we move forward.

The 3-nitro-2-phenyl-2*H*-chromene analogues revealed various differences in the effect of trypanosomatid growth inhibition between the *T. cruzi* infective form and the *T. brucei* bloodstream form (Figure 6b). Ethoxy and/or methoxy groups at positions R_1_ and R_2_ were observed to be very important for the growth inhibition of *T. brucei*. This became evident for the top seven potent anti-*T. brucei* compounds **1**, **3**, **4**, **5**, **6**, **8**, and **9**, all of which exhibited nanomolar efficacy (IC_50_ < 750 nM). In the case of *T. cruzi*, it appeared that the same substituents at R_1_ and R_2_ were only partially effective in aiding inhibition, because compounds **7** and **11**, which do not contain methoxy/ethoxy groups at R_1_/R_2_, also achieved a similar degree of *T. cruzi* growth inhibition as observed with compounds **3**, **4**, **5**, and **9**. More of the R_1_/R_2_ substituents being ethoxy/methoxy groups resulted in moderate to low inhibition in the *T. cruzi* parasite growth inhibition range (see Table 1 and Figure 1). Ortho halogen atoms positioned on the phenyl group were less important for *T. brucei* growth inhibition (see compounds **7**, **11**, **12**, and **13**, which resulted in lower inhibition of the set). On the other hand, ortho halogen atoms on the phenyl ring were very important for *T. cruzi* growth inhibition, as seen in compounds **7** and **11** (both exhibiting nanomolar efficacy in IC_50_). Regarding X_2_ and X_3_/R_3_ positions on the *2H*-chromene ring system, it was interesting to observe the two-bromine atom case at X_2_ and X_3_ for top inhibitor compounds against both trypanosomatid parasites. For example, compounds **5** and **7** for *T. cruzi* and compounds **5** and **6** for *T. brucei* exhibited this noteworthy antiparasitic effect. Compounds containing a methoxy group at position R_3_ and a bromine atom at X_2_ played a key role in the growth inhibition of both parasites, and a notable example was compound **3**.

## 3. Materials and Methods

### 3.1. Chemicals

Adenosine 5′-triphosphate disodium salt hydrate (≥99%), chlorophenol red-β-d-galactopyranoside (CPRG), β-nicotinamide adenine dinucleotide phosphate hydrate (NADP^+^), magnesium chloride hexahydrate (≥99%), d-(+)-glucose (≥99%), dimethyl sulfoxide (ACS reagent (≥99.9%)), d-glucose-6-phosphate sodium salt, *N*-(2-hydroxyethyl)piperazine-*N*′-(2-ethanesulfonic acid) (HEPES, ≥99.5%), imidazole (96.0%), Terrific broth modified, and triethanolamine were purchased from Sigma-Aldrich (St. Louis, MO, USA). Dulbecco’s modified Eagle’s medium (DMEM) was purchased from CellGro Technologies, LLC (Lincoln, NE, USA). *Leuconostoc mesenteroides* glucose-6-phosphate dehydrogenase (*Lm*G6PDH) was purchased from Worthington Biochemical Corporation (Lakewood, NJ, USA). NADPH tetrasodium salt (≥95%) was purchased from Enzo Life Sciences, Inc. (Farmingdale, NY, USA). For the 3-nitro-2-phenyl-*2H*-chromene analogues, compounds **1**–**7** were purchased from TimTec, LLC (Tampa, FL, USA), while compounds **8**–**13** were purchased from ChemDiv, Inc. (San Diego, CA, USA). Ethylenediaminetetraacetic acid (EDTA) tetrasodium salt hydrate (98%), Luria-Bertani (LB) broth, lysozyme (type VI), protease inhibitor tablets (EDTA-free), glycerol, and all other chemicals were purchased from Fisher Scientific (Hampton, NH, USA).

### 3.2. Recombinant Overexpression and Purification of TcGlcK

The recombinant overexpression of *Tc*GlcK in *Escherichia coli* followed by purification was similarly performed as previously described [27]. The heat shock method using *E. coli* strain BL21(DE3) (New England Biolabs, Inc.; Ipswich, MA, USA) for cell growth on LB-agar plates containing 50 µg/mL kanamycin was used for the transformation of the plasmid pET-*Tc*GlcK-xtal. Information regarding the gene synthesis and cloning was reported in our earlier work [27]. The starter cultures contained 5 mL of LB broth containing 50 µg/mL of kanamycin and were inoculated with a single colony of *E. coli* from the transformation step. The cultures were incubated at a constant temperature of 37 °C while shaking at 250 rpm for 8 h using a New Brunswick Scientific C24 incubator shaker. Six 2 L culture flasks with baffles containing 500 mL of Terrific broth supplemented with 0.4% (*v*/*v*) glycerol and 50 µg/mL of kanamycin were used in conjunction with the previous starting colonies incubated at 37 °C while shaking at 220 rpm for 16 h. The 500 mL cultures were then induced with isopropyl β-d-thiogalactoside to produce a final concentration of 1.0 mM. After adjusting the concentration, the cultures were incubated again for 24 h at 28–32 °C while shaking at 220 rpm. The *E. coli* cells were centrifuged at 7000 rpm in a Sorvall RC5C Plus centrifuge (SLA-1500 rotor) for 10 min at a temperature of 4 °C. The cell pellet recovered was stored in a −80 °C freezer overnight. The cell pellet was thawed to room temperature the following day and resuspended in a lysis buffer [50 mM HEPES (pH 7.0), 150 mM NaCl]. Cell lysis was performed on the cell suspension by adding ~500 mg of lyophilized lysozyme (type VI). The suspension was stirred for 1 h at a temperature of 4 °C before the addition of an EDTA-free protease inhibitor tablet. The cell lysate suspension underwent sonication for 30 min at a temperature of 0 °C (on ice/water) in a FS20 water-bath sonicator (Fisher Scientific). Once the sonication period was complete, the cell lysate was returned to a freezer to be stored at −20 °C overnight. The next morning, the cell lysate was once again thawed to room temperature before being centrifuged at 15,000 rpm in a Sorvall RC5C Plus centrifuge (SS-34 rotor) for 45 min at a temperature of 4 °C. The resulting supernatant was recovered and subsequently loaded onto a cobalt-nitriloacetic-acid–immobilized metal affinity chromatography column [1.5 cm (internal diameter) × 4.5 cm (bed height)] that had been pre-equilibrated with mobile phase A [50 mM HEPES (pH 7.0), 150 mM NaCl]. The column was initially washed with mobile phase A to elute any protein impurities before performing an isocratic elution step of 13% mobile phase B (note: the solution for 100% mobile phase B was 50 mM HEPES (pH 7.0), 300 mM NaCl, 150 mM imidazole). Once the UV absorbance (λ = 280 nm) on the FPLC’s chromatogram reached baseline, a second isocratic elution step from 13% to 100% mobile phase B was performed, in order to ensure the removal of more protein impurities.

The resulting fractions of *Tc*GlcK from the Co-NTA purification were combined and then concentrated to 5 mL using an Amicon Ultra-15 centrifugal concentrator (Fisher Scientific, Hampton, NH, USA) equipped with a YM-30 membrane (30 kDa molecular weight cutoff filter). The collected sample was loaded onto a 16/600 size-exclusion column (GE Healthcare, Pittsburgh, PA, USA) pre-equilibrated with mobile phase A [50 mM triethanolamine (pH 7.6), 150 mM NaCl]. Visual inspection of the SDS-PAGE on a 4–15% polyacrylamide gel showed that our fractions were >99% pure. Pure *Tc*GlcK fractions were pooled, and the concentration was adjusted to 1.0 mg/mL [ε_280_ = 33,710 M^−1^ cm^−1^ (*1*); M.W. = 42,174 g/mol (monomer of His-tagged *Tc*GlcK)].

### 3.3. Primary Screening Assay of TcGlcK and 3-Nitro-2-Phenyl-2H-Chromene Analogues

A primary screening assay was carried out for *Tc*GlcK with the 3-nitro-2-phenyl-*2H*-chromene analogues. The data was subsequently processed for respective K_i_ values in order to assess enzyme inhibition. Reaction mixtures were in a buffered solution (50 mM triethanolamine, 150 mM NaCl) and had a total volume of 206.5 μL that contained the following reagents at final assay concentrations: *Tc*GlcK (4.8 μg/mL), *Lm*G6PDH (6.1 μg/mL), NADP^+^ (0.54 mM), ATP (0.54 mM), MgCl_2_ (6.9 mM), DMSO (1.0% by vol.), d-glucose (variable concentrations, vide infra), and a 3-nitro-2-phenyl-*2H*-chromene analogue (variable concentrations, vide infra). The primary screening assay was performed with the substrate d-glucose in various concentrations of 0.4 mM, 0.6 mM, and 0.8 mM to produce Dixon plots with different slopes. Each of the 3-nitro-2-phenyl-*2H*-chromene analogues were originally prepared at a working concentration of 1.0 mM and were dissolved in 100% DMSO. The final assay concentrations of the analogue compounds (inhibitors) ranged from 0.0–10.0 μM. The analogue compounds were pre-incubated with the enzyme system a minimum of 10 min prior to the initiation of the assay. The enzymatic reaction was carried out at room temperature (22 °C; temperature optimum) using ATP to initiate the reaction, and termination was performed after 120 s (time optimum) by stopping the reaction with the addition of an EDTA (pH 8.0) solution, to a final concentration of 100 mM. *Tc*GlcK enzyme activity was analyzed for the production of NADPH. Each completed reaction mixture was added into a black, round-bottom microplate and scanned by a VANTAstar-F multi-mode microplate reader (BMG LABTECH, Inc.; Cary, NC, USA). The instrument was set to fluorescence mode and the wavelengths (λ_ex_ = 340 nm; λ_em_ = 485 nm) were set to observe the relative fluorescence units of NADPH [40]. The enzyme-inhibition kinetics K_i_ value determination and the mode of inhibition was determined using the Cornish–Bowden Dixon Plot analysis method [32]. Assays were performed singularly (*N* = 1) for compounds being initially screened; however, assays were performed in triplicate (*N* = 3) for compounds that inhibited *Tc*GlcK and resulted in observed K_i_ values less than 20 μM.

### 3.4. Confirmatory Assay

The stronger *Tc*GlcK inhibitors exhibiting K_i_ values less 20 μM in the *Tc*GlcK primary screening assay continued onto the confirmatory assay. Two criteria were examined in order to certify on-target confirmed hits: (a) the assessment of potential inhibition to the *Lm*G6PDH enzyme-coupled system, and (b) the precision of the assay (per inhibitor), performed in quadruplicate (*N* = 4). Compounds were tested in quadruplicate in a black, round-bottom 96-well microplate in which wells were filled with a buffered solution (50 mM triethanolamine, 150 mM NaCl) amounting to a total volume of 206.5 μL that contained the following reagents at final assay concentrations: *Lm*G6PDH (6.1 μg/mL), NADP^+^ (0.54 mM), ATP (0.54 mM), MgCl_2_ (6.9 mM), DMSO (1.0% by vol.), d-glucose-6-phosphate (0.80 mM), and a 3-nitro-2-phenyl-*2H*-chromene analogue (20.0 μM). The analogue compounds were pre-incubated with the enzyme system a minimum of 10 min prior to the initiation of the assay. The enzymatic reactions were initiated and terminated similarly as described in the *Tc*GlcK primary screen (*vide supra*). The production of NADPH was monitored using the VANTAstar-F multi-mode microplate reader set to fluorescent mode, λ_ex_ = 340 nm and λ_em_ = 485 nm, over a 120-s timeframe. Compounds acting as false-positives that would potentially inhibit the *Lm*G6PDH enzyme-coupled system were evaluated.

### 3.5. In Vitro T. cruzi Biological Assay

General methods for the preparation of in vitro cell cultures involving *T. cruzi* parasites and mammalian cells along with the exposure to inhibitors has previously been described by Andriani and colleagues [41]. *T. cruzi* (Tulahuen strain) that expresses β-galactosidase was harvested by centrifugation at 2500 rpm for 7 min. Parasites were rinsed twice with DMEM lacking phenol red and supplemented with 2% fetal bovine serum (FBS) and 1% penicillin—streptomycin—L-glutamine (PSG); the final concentrations of the PSG components were 100 U/mL penicillin, 0.1 mg/mL streptomycin, and 0.292 mg/mL L-glutamine. It was necessary to exclude phenol red since it interferes with the absorbance reading at λ = 590 nm. The parasites were centrifuged again for 7 min at 2500 rpm and the 50 mL tubes were carefully removed from the centrifuge. The tubes were placed on a rack with an incubator so that the trypomastigotes would move out of the pellet in a timeframe of 3–5 h. During this period, 100 μL of NIH-3T3 cells (50,000 cells) were plated per well in a transparent, clear, flat-bottom, and sterile 96-well microplate. This plate was placed in an incubator for 3 h at 37 °C to allow the cells to attach. The 3-nitro-2-phenyl-*2H*-chromene compounds (inhibitors) that were prepared as 10.0 mM stock solutions in 100% DMSO were thawed from −80 °C to room temperature. The compound solutions were diluted to their desired working concentrations, which ranged from 0.0781–10.0 μM. This was accomplished by serial dilution using a 2-fold dilution. A total of 100 μL of parasites was added to each well, which contained 50,000 trypomastigotes, and were incubated for 4 days. Using a multichannel pipette, 50.0 μL of the substrate solution [500 μM CPRG in phosphate buffered saline supplemented with 0.5% detergent NP-40] was added to each well of the 96-well microplate. The plate was placed into an incubator for 4 h followed by measuring absorbance readings at λ = 590 nm using a Victor Nivo multimode microplate reader (PerkinElmer, Inc.; Shelton, CT, USA). All measurements were performed in triplicate; absorbance values were proportional to parasite cell viability. IC_50_ determinations for percent growth inhibition were processed using GraphPad Prism 10.0. Amphotericin B was used as a positive control, the final solution concentration of which was 4.0 μM (prepared as a 270 μM stock solution). Four control conditions were carried out, as follows: (a) NIH-3T3 cells and parasites, (b) NIH-3T3 cells without parasites, (c) NIH-3T3 cells, parasites, and 4.0 μM amphotericin B, and (d) the medium alone.

### 3.6. In Vitro T. brucei Biological Assay

The in vitro cell culture preparation of *T. brucei brucei* (427 strain) trypomastigote bloodstream form followed by treatment with inhibitors has recently been described by Ajayi and colleagues [42]. The 3-nitro-2-phenyl-*2H*-chromene compounds (inhibitors) that were prepared as 10.0 mM stock solutions in 100% DMSO were thawed from −80 °C to room temperature. HMI-9 medium [Iscove’s modification of DMEM supplemented with 10% FBS, 10% serum plus (SAFC), 0.05 mM bathocuproinesulfonate, 1.5 mM L-cysteine, 1.0 mM hypoxanthine, 0.2 mM β-mercaptoethanol, 0.16 mM thymidine, and 1.0 mM pyruvate] was prepared. In a sterile, round-bottom, 96-well microplate, 100 μL of HMI-9 medium was added to each well. A volume of 2.0 μL of a given compound plus a volume of 98.0 μL of fresh media were both added to a well in the first row of the 96-well microplate; this was performed for each compound to be tested. Solutions were mixed with a micropipette followed by transfer of 100 μL to the next row and repeated. The compound solutions ranged from 0.0781–10.0 μM and this was accomplished by serial dilution using a 2-fold dilution. *T. brucei* parasites were centrifuged for 10 min at 900× *g* (without centrifuge breaks). The media was carefully discarded by aspiration and the parasites were resuspended in warm media. Parasites were counted in a Newbauer chamber. Afterwards, the parasites were diluted to 50,000 cells/mL (5000 cells/well) and 100 μL was added to each well of the 96-well microplate using a multichannel pipette. After 48 h, 20.0 μL of Alamar blue was added to each well and the plate was placed into an incubator for 4 h at 37 °C. Fluorescence readings were measured using a Victor Nivo multimode microplate reader (PerkinElmer, Inc.; Shelton, CT, USA) at λ_ex_ = 530 nm and λ_em_ = 590 nm. All measurements were performed in triplicate and IC_50_ determinations for percent growth inhibition were carried out using GraphPad Prism 10.0. The detergent SDS was used as a positive control and its final solution concentration was 0.5%. Three control experiments were performed, as follows: (a) parasites alone, (b) parasites and 0.5% SDS, and (c) the medium alone.

### 3.7. In Vitro Biological Assay for the Cytotoxicity of Mammalian NIH-3T3 Fibroblasts

The in vitro cell culturing of mammalian NIH-3T3 fibroblasts following treatment with inhibitors to assess for cytotoxicity has previously been described [17,41]. DMEM lacking phenol red and supplemented with 2% FBS and 1% PSG was warmed up. NIH-3T3 cells were trypsinized, as described in the cell culture protocol. When cells became detached, they were harvested in DMEM lacking phenol red and including 2% FBS and 1% PSG. The cells were centrifuged at 1000 rpm for 5 min, the medium was discarded, and the cells were resuspended in fresh DMEM lacking phenol red and including 2% FBS and 1% PSG. The cells were centrifuged at 1000 rpm for 5 min, resuspended in the medium, counted, and diluted to 500,000 cells/mL. Dilution was accomplished by transferring the stock number of cells into a sterile basin and 100 μL of cells was added to each well of a transparent, sterile, flat-bottom, 96-well microplate (50,000 cells/well) using a multichannel pipette. The plate was placed into the incubator for 3 h to allow cells to attach. The 3-nitro-2-phenyl-*2H*-chromene compounds were thawed from −80 °C to room temperature. The compounds were prepared as 10.0 mM stock solutions in 100% DMSO. A volume of 2.0 μL of a given compound plus a volume of 98.0 μL of fresh media were both added to a well in the first row of the 96-well microplate; this was performed for each compound to be tested. The solutions were mixed with a micropipette, followed by the transfer of 100 μL to the next row and repeated. The compound solutions ranged from 0.0139–30.3 μM and this was accomplished by serial dilution using a 3-fold dilution. After incubation for 4 days, 10.0 μL of Alamar blue was added to each well. Absorbance readings were measured using a Victor Nivo multimode microplate reader (PerkinElmer, Inc.; Shelton, CT, USA) at λ = 590 nm. All measurements were performed in triplicate and TC_50_ determinations for percent growth inhibition were carried out using GraphPad Prism 10.0. The final volume per well of cells and a given compound was 100 μL and this volume became 110 μL after the addition of Alamar blue. The detergent SDS was used as a positive control and its final solution concentration was 0.1%. Three controls were carried out, as follows: (a) NIH-3T3 cells alone, (b) perished NIH-3T3 cells (NIH-3T3 cells in the presence of 0.1% SDS), and (c) the medium alone.

## 4. Conclusions

The work described in this study revealed several important outcomes. First, it appears that the 3-nitro-2-phenyl-*2H*-chromene analogues might be inhibiting both *Tc*GlcK and *Tc*HxK, in parallel. This appears to explain why the compounds were more potent against *T. cruzi* in culture as compared to the enzymatic assays against *Tc*GlcK. Table 1 shows a ≥8-fold differential for 9 out of the 13 compounds in this regard. With this loss of correlation, the next topic to pursue is to determine whether or not *Tc*HxK is the other therapeutic drug target that these 3-nitro-2-phenyl-*2H*-chromene analogues are inhibiting. This would confirm the two-target hypothesis and rule out other off-targets in the parasite. One limitation we have found is that our laboratory has had difficulty over the years with the overexpression of recombinant *Tc*HxK and we are currently working at finding a resolution to this end. Second, various 3-nitro-2-phenyl-*2H*-chromene analogues from this study were determined to be potent anti-*T. brucei* compounds. We suspect that there is a two-target scenario for these investigational compounds in *T. brucei* as well, such as hexokinase isoenzymes I and II. We arrive to this conclusion since it is already known that *T. brucei* hexokinase I is a validated therapeutic drug target [36] and the *T. brucei* hexokinase isoenzymes most likely share a high degree of three-dimensional structural similarity to *Tc*HxK. Moreover, the virtual three-dimensional structural work by Omolabi and colleagues, in which they contended a theoretical binding site for two 3-nitro-2-phenyl-*2H*-chromene analogues on *Tc*HxK [30], seems reasonable. However, we believe that in order to move forward, their computational work should be investigated experimentally to validate their binding site hypothesis. It is plausible that the same binding site exists on the *T. brucei* hexokinases. Finally, the results presented herein indicate that an extensive screening library of 3-nitro-2-phenyl-*2H*-chromene compounds should be developed, in order to arrive at even stronger antitrypanosomal agents.

## Figures and Tables

**Table 1:** Effect of 3-nitro-2-phenyl-*2H*-chromene analogues on the inhibition of *Tc*GlcK and on the biological activity against *T. cruzi*, *T. brucei*, and NIH-3T3 fibroblast cytotoxicity.

Compound	*Tc*GlcK K_i_ (µM) *^a^*	*Lm*G6PDH Inh20 (%) *^c,d^*	*T. cruzi* IC_50_ (µM) *^b,e^*	*T. brucei* IC_50_ (µM) *^b,f^*	NIH-3T3 TC_50_ (µM) *^b,g^*
**1**	6.2 ± 1.2 *^b^*	15.4 ± 0.1	1.49 ± 0.07	0.61 ± 0.03	16.0 ± 1.84
**2**	46	0.0 ± 0.0	1.44 ± 0.07	1.19 ± 0.04	22.2 ± 0.33
**3**	24	0.0 ± 0.0	0.96 ± 0.02	0.52 ± 0.05	17.5 ± 1.90
**4**	5.5 ± 1.8 *^b^*	12.1 ± 0.0	1.31 ± 0.15	0.44 ± 0.03	16.4 ± 1.91
**5**	35	0.0 ± 0.0	0.64 ± 0.23	0.42 ± 0.04	19.5 ± 0.51
**6**	6.1	16.1 ± 0.0	2.56 ± 0.26	0.36 ± 0.06	18.1 ± 0.24
**7**	50	0.0 ± 0.0	0.64 ± 0.02	0.90 ± 0.33	11.9 ± 0.51
**8**	30	0.0 ± 0.0	1.85 ± 0.15	0.74 ± 0.14	26.1 ± 0.57
**9**	12.3 ± 5.5 *^b^*	10.1 ± 0.1	0.64 ± 0.17	0.66 ± 0.05	17.5 ± 1.60
**10**	23	0.0 ± 0.0	2.56 ± 0.26	1.12 ± 0.30	9.45 ± 2.20
**11**	20.0 ± 4.3 *^b^*	15.0 ± 0.1	0.77 ± 0.02	0.86 ± 0.20	4.12 ± 1.95
**12**	14	8.6 ± 0.1	7.07 ± 1.19	1.67 ± 0.06	10.9 ± 1.55
**13**	25	0.0 ± 0.0	3.08 ± 0.08	1.28 ± 0.10	nd *^h^*

*^a^* Number of replicates, *N* = 1, unless otherwise stated. *^b^* Number of replicates, *N* = 3. *^c^* Number of replicates, *N* = 4. *^d^* Inh20 refers to the percentage of inhibition of 20 µM of the compound in comparison to controls. *^e^* in vitro *T. cruzi* (Tulahuen strain) infective form growth inhibition co-cultured in NIH-3T3 fibroblasts (see Figure S1 in the Supplementary Information section). *^f^* in vitro *T. brucei brucei* (427 strain) infective bloodstream form growth inhibition (see Figure S2 in the Supplementary Information section). *^g^* in vitro NIH-3T3 fibroblast growth inhibition for the evaluation of host cell toxicity (see Figure S3 in the Supplementary Information section). *^h^* nd, “not determined”.

## Data Availability

Data pertaining to key findings is contained within the article and the Supplementary Information section.

## Supplementary Materials

The following supporting information can be downloaded at: https://www.mdpi.com/article/10.3390/ijms25084319/s1. Reference [43] is cited in the Supplementary Information section.

## Abbreviations

CPRG, chlorophenol red-β-d-galactopyranoside; DMEM, Dulbecco’s modified Eagle’s medium; DMSO, dimethyl sulfoxide; EDTA, ethylenediaminetetraacetic acid; FBS, fetal bovine serum; G6P, d-glucose-6-phosphate; HEPES, *N*-(2-hydroxyethyl)piperazine-*N*′-(2-ethanesulfonic acid); HMI-9, Hirumi’s modified Iscove’s medium 9; HTS, high-throughput screening; LB, Luria-Bertani; *Lm*G6PDH, *Leuconostoc mesenteroides* glucose-6-phosphate dehydrogenase; NADP^+^, β-nicotinamide adenine dinucleotide phosphate; NADPH, β-nicotinamide adenine dinucleotide phosphate (reduced form); PPP, pentose phosphate pathway; PSG, penicillin—streptomycin—l-glutamine; SAR, structure–activity relationship; SDS, sodium dodecyl sulfate; SDS-PAGE, sodium dodecyl sulfate–polyacrylamide gel electrophoresis; *Tc*GlcK, *Trypanosoma cruzi* glucokinase; *Tc*HxK, *Trypanosoma cruzi* hexokinase.
