# Supplementary material for: Discovery of Strong 3-Nitro-2-Phenyl-2H-Chromene Analogues as Antitrypanosomal Agents and Inhibitors of Trypanosoma cruzi Glucokinase
Source: Int J Mol Sci. 2024 Apr 13;25(8):4319. doi: 10.3390/ijms25084319 (PMC11050443; doi:10.3390/ijms25084319)
Supplement: Supplementary file 1 [file ijms-25-04319-s001.zip › ijms-2935543-supplementary.pdf]

Supplementary Information

Discovery of Strong 3-Nitro-2-Phenyl-*2H*-Chromene Analogues as Antitrypanosomal Agents and Inhibitors of *Trypanosoma cruzi* Glucokinase

Shane M. Carey,[a,c] *Destiny M. O'Neill,* [a,c] *Garrett B. Conner,*[a] *Julian Sherman,*[b]

Ana Rodriguez,[b] and *Edward L. D'Antonio* [a,*]

[a] Department of Natural Sciences, University of South Carolina Beaufort, 1 University Boulevard, Bluffton, South Carolina 29909, USA

[b] Department of Microbiology, New York University School of Medicine, 430 East 29th Street, New York, New York 10016, USA

[c] Authors contributed equally to this work.

* To whom correspondence should be addressed:

Prof. Edward L. D'Antonio, Ph.D.
Department of Natural Sciences
University of South Carolina Beaufort
1 University Boulevard
Bluffton, South Carolina 29909
United States of America
Tel.: +1 843-208-8101
E-mail: edantonio@uscb.edu

Table of Contents

I. Chemical Structures of the 3-Nitro-2-Phenyl-*2H*-Chromene Analogues

a. **Table S1.** Systematic Names of Compounds **1** – **13**…………………….…………**S3**

b. **Table S2.** SMILES Codes of Compounds **1** – **13**.………………………………**S4**

II. Biological Assays

a. **Figure S1.** *In vitro* dose – response of compound activity (**1** – **13**) on *Trypanosoma cruzi* (Tulahuen strain) infective form co-cultured in mammalian NIH-3T3 fibroblasts………...…………………………………………………...……………**S5**

b. **Figure S2.** *In vitro* dose – response of compound activity (**1** – **13**) on *Trypanosoma brucei brucei* (427 strain) bloodstream form……………………………………..**S6**

c. **Figure S3.** *In vitro* dose – response of compound activity (**1** – **12**) on mammalian NIH-3T3 fibroblasts in order to assess cytotoxicity………………….…………**S7**

III. Reference……………………………………………………………………………**S8**

I. Chemical Structures of the 3-Nitro-2-Phenyl-2H-Chromene Analogues

a. Systematic Names of Compounds 1 – 13.

Table S1. The systematic names for 3-nitro-2-phenyl-*2H*-chromene analogues **1 – 13**.

Compound	Systematic Name[a]
**1**	6-Bromo-2-(4-ethoxy-3-methoxyphenyl)-3-nitro-*2H*-chromene
**2**	6-Bromo-2-(3,4-diethoxyphenyl)-3-nitro-*2H*-chromene
**3**	6-Bromo-2-(3,4-dimethoxyphenyl)-8-methoxy-3-nitro-*2H*-chromene
**4**	6-Bromo-2-(3,4-dimethoxyphenyl)-3-nitro-*2H*-chromene
**5**	6,8-Dibromo-2-(3,4-dimethoxyphenyl)-3-nitro-*2H*-chromene
**6**	6,8-Dibromo-2-(4-ethoxy-3-methoxyphenyl)-3-nitro-*2H*-chromene
**7**	2-(2,6-Dichlorophenyl)-6,8-dibromo-3-nitro-*2H*-chromene
**8**	6-Bromo-2-(4-ethoxy-3-methoxyphenyl)-8-methoxy-3-nitro-*2H*-chromene
**9**	6,8-Dichloro-2-(3,4-diethoxyphenyl)-3-nitro-*2H*-chromene
**10**	2-(3,4-Diethoxyphenyl)-3-nitro-*2H*-chromene
**11**	6-Bromo-2-(2-chlorophenyl)-3-nitro-*2H*-chromene
**12**	2-(2-Chlorophenyl)-3-nitro-*2H*-chromene
**13**	2-(2,6-Dichlorophenyl)-3-nitro-*2H*-chromene

[a] Systematic names were generated through *ChemDraw Ultra 12.0* using a sketch of the compound.

b. SMILES Codes for Compounds 1 – 13.

Table S2. SMILES codes for the 3-nitro-2-phenyl-*2H*-chromene analogues (**1 – 13**).

Compound	SMILES Code[a-c]
**1**	BrC=1C=C2C=C(C(OC2=CC1)C1=CC(=C(C=C1)OCC)OC)[N+](=O)[O-]
**2**	BrC=1C=C2C=C(C(OC2=CC1)C1=CC(=C(C=C1)OCC)OCC)[N+](=O)[O-]
**3**	BrC=1C=C2C=C(C(OC2=C(C1)OC)C1=CC(=C(C=C1)OC)OC)[N+](=O)[O-]
**4**	BrC=1C=C2C=C(C(OC2=CC1)C1=CC(=C(C=C1)OC)OC)[N+](=O)[O-]
**5**	BrC=1C=C2C=C(C(OC2=C(C1)Br)C1=CC(=C(C=C1)OC)OC)[N+](=O)[O-]
**6**	BrC=1C=C2C=C(C(OC2=C(C1)Br)C1=CC(=C(C=C1)OCC)OC)[N+](=O)[O-]
**7**	ClC1=C(C(=CC=C1)Cl)C1OC2=C(C=C(C=C2C=C1[N+](=O)[O-])Br)Br
**8**	BrC=1C=C2C=C(C(OC2=C(C1)OC)C1=CC(=C(C=C1)OCC)OC)[N+](=O)[O-]
**9**	ClC=1C=C2C=C(C(OC2=C(C1)Cl)C1=CC(=C(C=C1)OCC)OCC)[N+](=O)[O-]
**10**	C(C)OC=1C=C(C=CC1OCC)C1OC2=CC=CC=C2C=C1[N+](=O)[O-]
**11**	BrC=1C=C2C=C(C(OC2=CC1)C1=C(C=CC=C1)Cl)[N+](=O)[O-]
**12**	ClC1=C(C=CC=C1)C1OC2=CC=CC=C2C=C1[N+](=O)[O-]
**13**	ClC1=C(C(=CC=C1)Cl)C1OC2=CC=CC=C2C=C1[N+](=O)[O-]

[a] SMILES codes were generated through *OPSIN* (https://opsin.ch.cam.ac.uk) by entering the systematic names (see **Table S1**).

[b] Ref. Lowe et al. *(1)*.

[c] Structures of compounds can be visualized by entering SMILES codes into the program *PubChem Sketcher v2.4* (https://pubchem.ncbi.nlm.nih.gov//edit3/index.html).

II. Biological Assays.

a. Figure S1. *In vitro* dose – response of compound activity (**1** – **13**) on *Trypanosoma cruzi* (Tulahuen strain) infective form co-cultured in mammalian NIH-3T3 fibroblasts. Data was collected in triplicate and the results are presented as IC_{50} plots shown as percent growth inhibition as a function of log ([compound]).

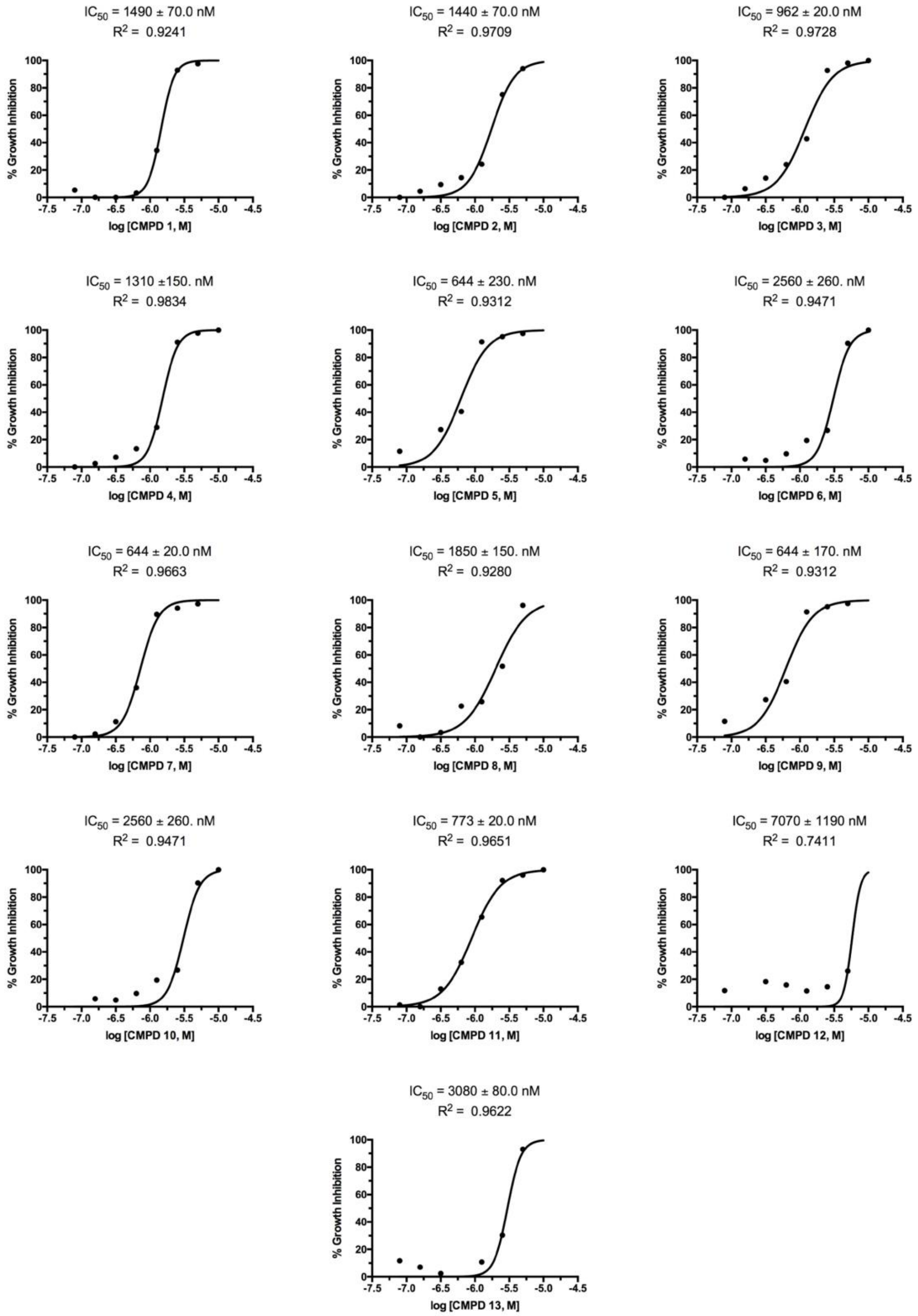

b. **Figure S2.** *In vitro* dose – response of compound activity (**1** – **13**) on *Trypanosoma brucei brucei* (427 strain) bloodstream form. Data was collected in triplicate and the results are presented as IC_{50} plots shown as percent growth inhibition as a function of log ([compound]).

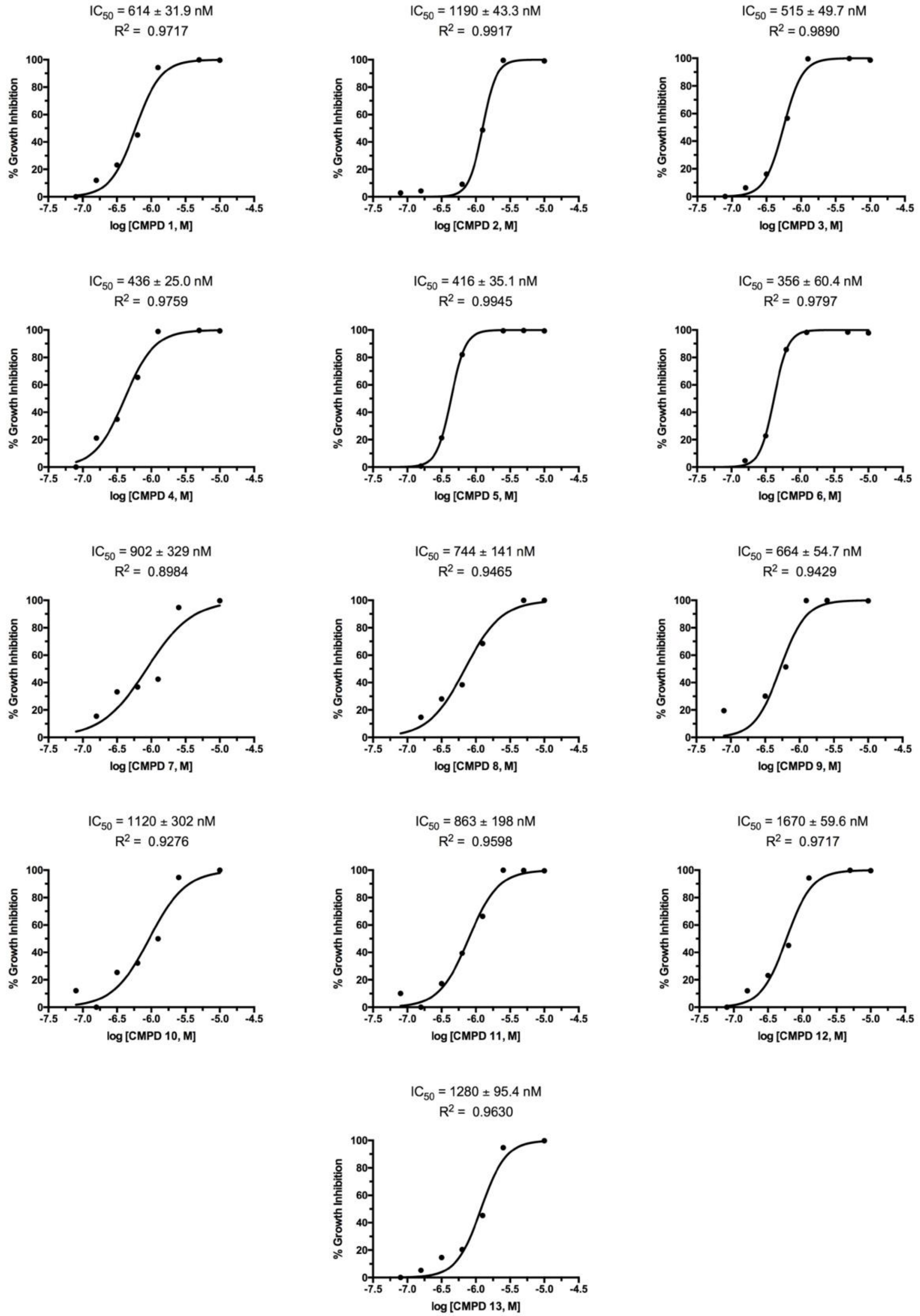

c. Figure S3. *In vitro* dose – response of compound activity (**1** – **12**) on mammalian NIH-3T3 fibroblasts in order to assess cytotoxicity. Data was collected in triplicate and the results are presented as TC_{50} plots shown as percent growth inhibition as a function of log ([compound]). Note: Compound **13** was excluded due a poor fit to the sigmoidal curve.

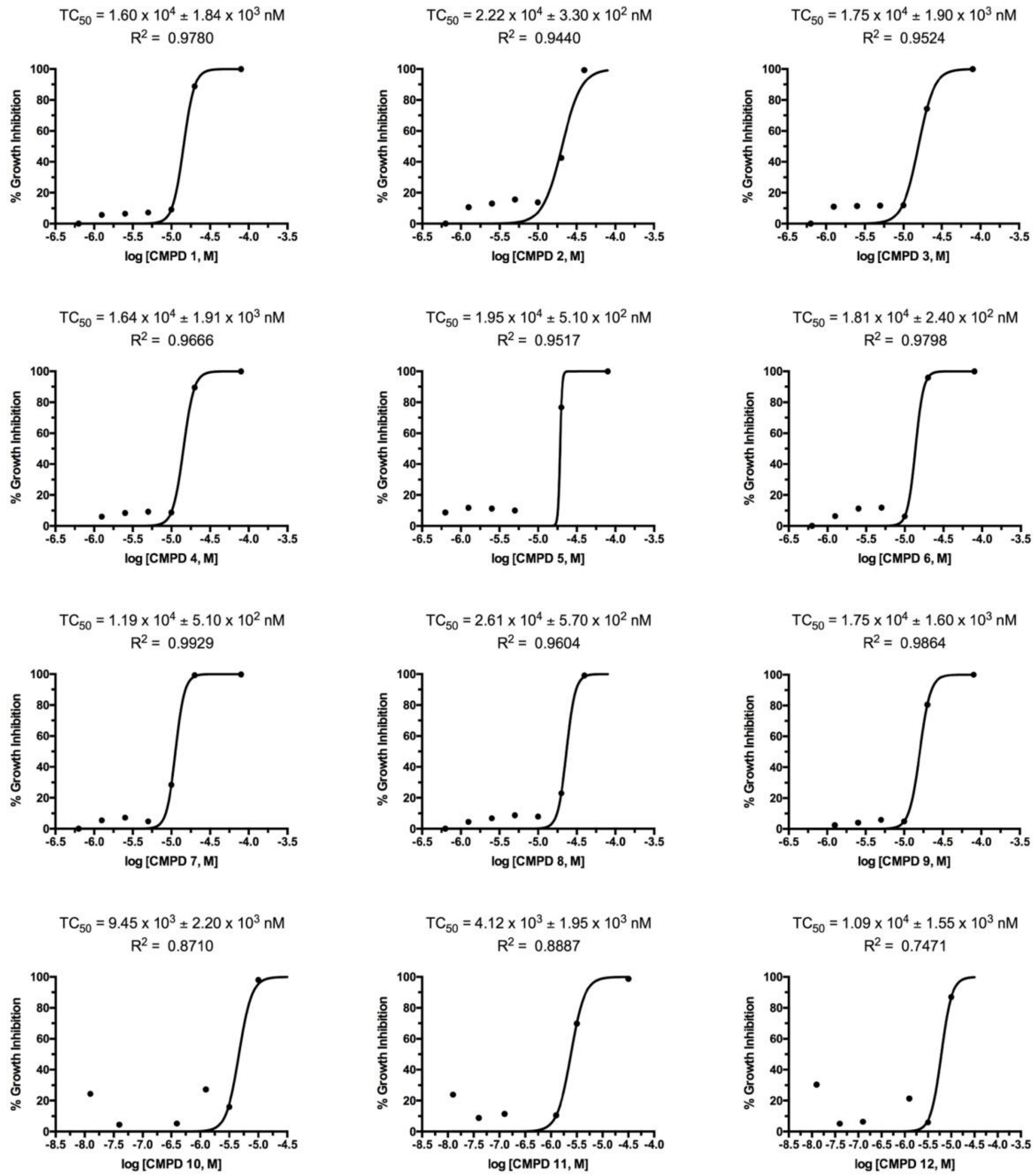

Reference

([1.) Lowe, D. M., Corbett, P. T., Murray-Rust, P., and Glen, R. C. (**2011**) Chemical name to structure: OPSIN, an open source solution, *J. Chem. Inf. Model 51*, 739-753.
